# The Relationships between Hip and Knee Extensor Cross-Sectional Area, Strength, Power, and Potentiation Characteristics

**DOI:** 10.3390/sports5030066

**Published:** 2017-09-05

**Authors:** Timothy J. Suchomel, Michael H. Stone

**Affiliations:** 1 Department of Human Movement Sciences, Carroll University, Waukesha, WI 53186, USA; 2 Department of Exercise and Sport Sciences, Center of Excellence for Sport Science and Coach Education, East Tennessee State University, Johnson City, TN 37614, USA; stonem@etsu.edu

**Keywords:** postactivation potentiation, back squat, half-squat, squat jump, ultrasound, vastus lateralis, biceps femoris

## Abstract

The purpose of this study was to examine the relationships between muscle cross-sectional area (CSA), maximal strength, power output, and maximum potentiation characteristics. The vastus lateralis and biceps femoris CSA, one repetition maximum (1RM) back squat, 1RM concentric-only half-squat (COHS) strength, static jump power output, and maximum potentiation characteristics of 17 resistance-trained men was assessed during several testing sessions. Pearson’s correlation coefficients were used to examine the relationships between CSA, strength, power output, and maximum potentiation measures. Moderate-to-strong relationships existed between CSA and strength measures (*r* = 0.462–0.643) as well as power output (*r* = 0.396–0.683). In addition, moderate-to-strong relationships existed between strength and power output (*r* = 0.407–0.548), while trivial relationships existed between strength and maximum potentiation (*r* = −0.013–0.149). Finally, small negative relationships existed between CSA and maximum potentiation measures (*r* = −0.229–−0.239). The results of the current study provide evidence of the interplay between muscle CSA, strength, power, and potentiation. Vastus lateralis and biceps femoris CSA may positively influence an individual’s back squat and COHS maximal strength and squat jump peak power; however, muscle CSA and absolute strength measures may not contribute to an individual’s potentiation capacity. Practitioners may consider implementing resistance training strategies that improve vastus lateralis and biceps femoris size in order to benefit back squat and COHS strength. Furthermore, implementing squatting variations—both full and partial—may benefit jumping performance.

## 1. Introduction

A sequenced progression of training phases can result in the enhanced development of fitness characteristics that contribute to an individual’s overall performance. Previous literature has indicated that increases in work capacity and the cross-sectional area (CSA) of the involved musculature during an initial strength-endurance phase may enhance an individual’s ability to increase their muscular strength during subsequent training phases [1,2,3]. Furthermore, increases in an individual’s force production capacity and rate of force development during maximal strength training phases may then lead to an increased ability to demonstrate greater power output [4,5], as well as additional abilities such as postactivation potentiation (PAP) [4,6,7,8]. Additional research supports this notion as muscle CSA was shown to be a strong predictor of the variance in muscular strength and neuromuscular performance (maximal vertical jump performance) in adult men and women [9]. Thus, it may be argued that muscle CSA serves as an important foundation on which both muscular strength and power may be built.

A recent review reported strong relationships between measures of muscular strength and a number of performance characteristics including rate of force development, power output, general sport skills (jumping, sprinting, and change of direction), specific sport tasks, and PAP [4]. Although an abundance of literature has examined relationships between back squat strength and performance, a paucity of literature has examined the relationships between squats performed from different depths and performance. Given that muscle activation [10,11,12] and relative muscular effort [13] may vary with squat depth, it is important to examine these relationships to determine how muscular strength in various positions affects performance. Furthermore, considering that hip and knee extensors are frequently trained during resistance training programs that include squatting variations, it is important to understand how the characteristics of these muscles (e.g., CSA) contribute to muscular strength during these movements. From a practical standpoint, these analyses would provide further information regarding the possible implementation of partial squats into strength training programs as well as provide insight into the relationships between hip and knee extensor size and maximal squat and partial squat strength.

Results of previous studies indicate that participants with greater lower extremity absolute [14,15,16] and/or relative strength [17,18,19] may produce greater magnitudes of PAP compared to their weaker counterparts. Additional research demonstrated that one may achieve greater potentiation after increasing their strength [20]. While it appears that muscular strength is a strong predictor of PAP, it is possible that an individual’s muscle CSA may also be a predictor of PAP, given its influence on muscular strength. A recent study by Seitz and colleagues [21] indicated that quadriceps CSA was strongly correlated (*r* = 0.68) with maximal voluntary PAP response during isokinetic knee extensions. Although the previous study provides some indication of the relationships between muscle CSA and PAP, it should be noted that the vast majority of potentiation literature has sought to enhance the performance of more complex tasks such as vertical jumping. Thus, it appears that further research is warranted to provide sport scientists and practitioners with information on how muscle CSA relates to maximal PAP during a multi-joint movement. These findings may have practical applications given the attention that potentiation complexes have received within the strength and conditioning literature. Therefore, the purpose of this study was to examine the relationships between muscle CSA, maximal strength, power output, and maximum PAP. Based on the extant literature, it was hypothesized that strong relationships would exist between muscle CSA, strength, and power output and that small relationships would exist between CSA and maximum PAP.

## 2. Materials and Methods

### 2.1. Participants

Seventeen resistance-trained males (age = 24.2 ± 4.2 years, height = 180.1 ± 8.6 cm, body mass (BM) = 86.5 ± 9.2 kg, one repetition maximum (1RM) back squat = 164.7 ± 29.9 kg, 1RM concentric-only half-squat (COHS) = 194.6 ± 28.2 kg, relative back squat 1RM = 1.9 ± 0.3 kg/kg BM, relative COHS 1RM = 2.3 ± 0.3 kg/kg BM) who regularly trained with the back squat exercise volunteered to participate in this study. All participants reported that they were performing ≥ three resistance sessions per week. Prior to testing, all participants read and signed a written informed consent form. This study was approved by the University’s Institutional Review Board.

### 2.2. 1RM Back Squat Testing Session

The purpose of the 1RM back squat testing session was to establish each participant’s 1RM back squat and determine the starting position for the 1RM COHS testing session. Prior to testing, each participant performed a standardized general warm-up (two minutes of stationary cycling at 50 W at approximately 70 rpm). This was followed by a dynamic warm-up that included dynamic stretches covering a distance of 10 m (e.g., forward walking lunge, straight leg march, etc.) and five repetitions each of slow bodyweight squats and fast bodyweight squats. Two minutes of rest were provided following the warm-up before the participant started the 1RM back squat test protocol as outlined by previous research [22]. Briefly, each participant performed 5, 5, 3, and 1 warm-up repetition(s) at 30, 50, 70, and 90% of their self-determined 1RM, respectively. Following the warm-up back squat sets, participants completed maximal back squat attempts, with four minutes of recovery between attempts, at progressively increasing loads until a failed attempt occurred. The loads were determined by the primary investigator and research assistants based on the previous 1RM attempt. A minimum 2.5 kg increase was required and all participants achieved their 1RM back squat in four attempts or fewer. All back squat repetitions were performed to a depth where the participant’s hip crease dropped below their patella.

After the completion of the 1RM back squat, participants were provided with a self-selected rest period. Following the rest period, each participant squatted with a 20 kg barbell to a knee angle of 90° in order to determine the safety bar height for the 1RM COHS that would be performed during the following 1RM COHS session. The knee angle was verified through the use of a manual goniometer and the safety bar heights were adjusted accordingly. After the safety bars were adjusted, the participant positioned themself under the barbell to confirm that their position for the COHS 1RM test was correct.

### 2.3. 1RM Concentric-Only Half-Squat Testing Session

Each participant returned for the 1RM COHS testing session one week following the 1RM back squat session. The 1RM COHS testing session was used to determine the loads that would be used during the potentiation testing session, and to familiarize the participants with the testing conditions. Prior to testing, participants performed the same warm-up protocol as described above. Similar to the 1RM back squat testing session, the participant began performing warm-up COHS repetitions following a two minute rest period. Warm-up sets of 5, 5, 3, and 1 repetition(s) were performed at 30, 50, 70, and 90% of the participant’s estimated 1RM COHS as described by previous research [22]. The 1RM COHS warm-up loads were based on previous pilot testing that indicated that the 1RM COHS was approximately 1.2× the participant’s 1RM back squat. Following the final warm-up set, participants performed maximal COHS attempts, with four minutes of recovery between attempts, at progressively increasing loads until a failed attempt occurred. All COHS repetitions were performed with the barbell resting on the safety pins of the squat rack with the participant starting with a 90° knee angle. The participants then used a concentric-only muscle action to finish each repetition [22]. Each participant’s 1RM COHS was determined in four attempts or fewer.

### 2.4. Potentiation Testing Session

One week following the 1RM COHS session, participants arrived for the potentiation session. Participants completed the same general warm-up procedures described above before receiving final instructions before they completed their baseline static jumps (SJ) on the force plates. Following warm-up SJs at 50% and 75% of their perceived maximum effort, participants performed two maximal effort SJs with one minute of rest between jumps. Participants then began the potentiation protocol two minutes following their baseline jumps. The potentiation protocol was based on previous research [17,22,23] and consisted of five COHS repetitions at 30%, three repetitions at 50%, three repetitions at 70%, and two repetitions at 90% 1RM of the participant’s previously established 1RM COHS. All COHS were performed in a ballistic manner as previously described [17,22,23]. Two minutes of rest were provided following the sets at 30% and 50% 1RM and four minutes were provided following the set at 70% 1RM. Immediately following the final COHS repetition, each participant walked out of the squat rack and stepped onto the force plates. The participant was then instructed to squat down to the “ready position” (i.e., 90° knee angle) and received a countdown (i.e., “3, 2, 1, jump!”). The participants then performed a SJ using a concentric-only movement to jump as high as possible while holding a near weightless (<1 kg) PVC pipe on their upper back, similar to a high bar back squat position. Subsequent SJs were performed in the same manner every minute up to 10 min following the completion of the potentiation protocol, similar to previous research [17].

### 2.5. Ultrasonography

Prior to performing the warm-up and 1RM back squat protocol, CSA measurements of the participants’ right vastus lateralis (VL) and biceps femoris (BF) muscles were assessed using a linear probe scanning head with a 3.4–10.8 MHz bandwidth range (LOGIQ P6, GE Healthcare, Wauwatosa, WI, USA). The probe was coated with a water soluble transmission gel (Aquasonic 100 ultrasound transmission gel, Parker Laboratories, Inc., Fairfield, NJ, USA) and positioned on the surface of the skin to provide acoustic contact without depressing the dermal layer to collect an image. The CSA images for the VL and BF were obtained using a sweep of the muscle in the extended field of view mode with the gain set to 50 dB and an image depth to 5 cm. For the VL CSA measurements, participants laid on an athletic training table on their left side with their legs together and relaxed with 15° of knee flexion as measured by a manual goniometer [24]. For the BF CSA measurements, participants laid in a prone position with their feet hanging off the end of the athletic training table. The anatomical location for all CSA measurements was standardized for all participants. VL CSA was measured at 50% of the distance between the greater trochanter and the lateral condyle of the tibia. BF CSA was measured at 50% of the distance between the ischial tuberosity and the posterior aspect of the fibular head.

### 2.6. Data and Statistical Analyses

All SJ repetitions were performed on a dual force plate setup (two separate 45.5 cm × 91 cm force plates; RoughDeck HP, Rice Lake, WI, USA) sampling at 1000 Hz. The SJ data were collected and analyzed using a customized LabVIEW program (2012 Version, National Instruments Co., Austin, TX, USA). Voltage data obtained from the force plates were filtered using a digital low-pass Butterworth filter with a cutoff frequency of 10 Hz in order to minimize noise within the signal [23]. Static jump peak power (PP) was calculated as the product of force and velocity and as the greatest instantaneous value from the raw power-time data. The average values of PP were calculated between the two baseline repetitions and compared with the values obtained during the SJs performed at each post-stimulus rest interval (i.e., immediately and 1–10 min) during the potentiation session. The percentage improvement (i.e., potentiation) in group SJ PP was determined at each rest interval and the greatest mean improvement was used for the correlation analysis. Baseline SJs were used for the reliability analysis. Three CSA measurements were taken for both the VL and BF. Each trial was used for the reliability analysis and the average CSA was used for further statistical analysis.

Two-way random effects mixed model intraclass correlation coefficients (ICC) and typical error (TE) expressed as a coefficient of variation percentage were used to assess inter-trial reliability. A paired-samples *t*-test was used to examine the differences between baseline SJ PP and the time interval in which the maximum PP potentiation occurred. In addition to 95% confidence intervals (CI), Cohen’s d effect sizes were used to indicate the practical significance between baseline PP and maximum PP potentiation. Pearson’s correlation coefficients (*r*) were used to examine the relationships between VL and BF CSA, 1RM back squat, 1RM COHS, SJ PP, and maximum percent PP potentiation. Based on the current sample size, it was determined that a correlation of 0.48 was needed to demonstrate a statistically significant relationship. Effect size and correlation magnitudes were interpreted based on previous scales displayed by Hopkins [25]. All statistical analyses were performed using SPSS 23 (IBM, Armonk, NY, USA) and statistical significance was set at *p* ≤ 0.05.

## 3. Results

The inter-trial reliability ICC for VL and BF CSA were both 0.99 and the typical error percentages were 2.5% and 2.9%, respectively. In addition, the ICC and typical error percentage for SJ PP were 0.98 and 2.0%, respectively. The descriptive data are displayed in Table 1.

### 3.1. Static Jump Potentiation

**Figure 1:**
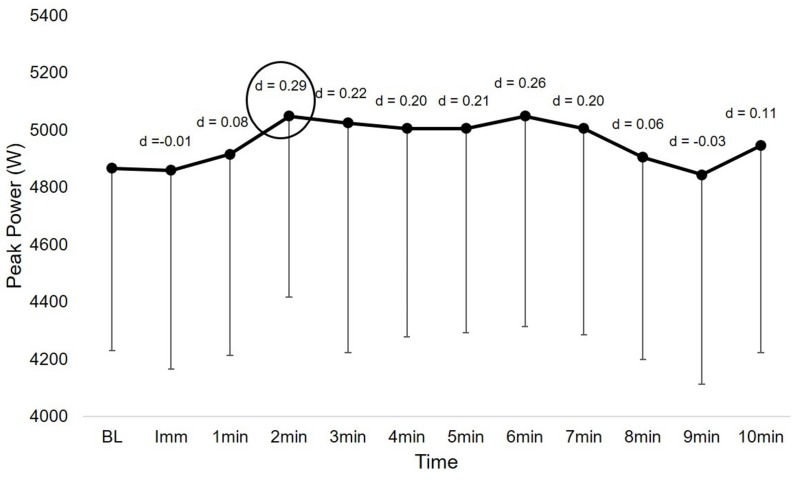
Temporal profile of peak power potentiation with Cohen’s d effect size differences from baseline (BL). Notes: Imm = immediately (i.e., ~15 s following potentiation stimulus); circle = rest interval that demonstrated the greatest potentiation, used for statistical comparison with baseline performance.

Peak PAP for the participants occurred at two minutes following the potentiation stimulus. Statistically significant differences for SJ PP existed between baseline and PP at two minutes post-stimulus (*t* = 4.233, *p* = 0.001, d = 0.29, CI = 90.6–272.2). Figure 1 displays the change in SJ PP at each time point with Cohen’s d effect sizes indicating the difference in performance compared to baseline.

### 3.2. CSA, Strength, Power, and Potentiation Relationships

Large positive relationships were present between VL and BF CSA measurements and strength measurements (i.e., 1RM back squat and 1RM COHS), with the exception of a moderate relationship existing between BF CSA and 1RM COHS. In contrast, small negative relationships existed between muscle CSA measurements and maximum potentiation. Large and moderate positive relationships existed between 1RM back squat and 1RM COHS with SJ PP, respectively. In contrast, trivial relationships existed between both strength measures and maximum potentiation; however, follow-up analyses between relative back squat and COHS 1RM and maximum potentiation displayed larger relationships (*r* = 0.196 and *r* = 0.398, respectively). Finally, a small negative relationship existed between SJ PP and maximum potentiation. The relationships between muscle CSA, strength, power, and maximum potentiation measures are displayed in Table 2.

## 4. Discussion

The primary findings regarding the relationships with CSA measurements are as follows: first, moderate-large relationships existed between VL and BF CSA and maximal strength measures; second, moderate-large relationships existed between VL and BF CSA measures and SJ PP; third, trivial relationships existed between strength and maximum PAP measures; and finally, small negative relationships existed between VL and BF CSA measures and maximum PAP.

Classic work from Stone et al. [26], Minetti [1], and Zamparo et al. [2] suggests that increasing muscle CSA, particularly in a task specific manner, can lead to an increased ability to improve the muscle’s maximal force production capacity (i.e., strength) in the same or similar tasks. From a physiological perspective, an increase in muscle CSA leads to an improved capacity to produce force due to the addition of newly formed sarcomeres. The addition of sarcomeres then increases the number of potential interactions between actin and myosin within the sarcomere (i.e., cross-bridges), causing an increase in the potential force that may be produced by a given muscle. This idea is supported by previous research that displayed increases in muscle fiber pennation angles with increases in muscle hypertrophy [27]. Our results support the previous literature as moderate-to-large relationships existed between the participants’ VL and BF CSA and multiple measures of maximal strength. Thus, it appears that a sequenced progression of training phases may allow an earlier phase of training to lay the foundation to potentiate or enhance the subsequent phase(s) of training [3]. However, it should be noted that the constraints of each maximal strength test may have affected the magnitude of some of the relationships. For example, both the VL and BF CSA measures displayed similar large relationships with the participants’ back squat strength. In contrast, while a large relationship was displayed between VL CSA and COHS strength, only a moderate relationship was displayed with BF CSA. Although not measured in the current study, previous literature indicated that greater squat depths increased the relative muscular effort of both hip and knee extensors [13]. Thus, it is possible that the relative effort of the BF may have been reduced during the COHS, due to a shallower squat, which may have contributed to a smaller relationship compared to that of the VL.

Muscle CSA measures displayed moderate-to-large relationships with SJ PP, which is similar to previous research [28,29]. It is interesting to note that the relationship between the BF CSA and SJ PP produced a stronger correlation compared to VL CSA and SJ PP. However, these differences may be explained by the fiber arrangement of each muscle. Although not measured in the current study, previous research indicated that the hamstring muscles possess a greater number of sarcomeres in series compared to the quadriceps muscles [30]. Based on this fiber arrangement and the length of the muscles, the hamstrings may result in a 30% greater shortening velocity compared to the quadricep muscles [30], which may ultimately result in greater magnitudes of power output. In contrast, the quadriceps may produce approximately 40% greater muscle tension compared to the hamstrings [30], likely the result of a larger number of sarcomeres in parallel. The findings of the current study provide further support that muscle CSA may serve as a foundation for both muscle strength and power characteristics.

While moderate-to-strong relationships existed between muscle CSA, strength, and power output, the same cannot be said for the relationships between muscle CSA and maximum PAP. Small negative relationships were displayed between both VL and BF CSA and maximum PAP within the current study. Our results are in contrast to previous literature that displayed a large positive relationship (*r* = 0.68) between quadriceps CSA and maximal voluntary PAP response during isokinetic knee extensions [21]. Although conflicting evidence, the complexity of the potentiation task, in this case a SJ, must be taken into consideration. Previous literature discussed how both the characteristics of the participant as well as the potentiation complex may affect whether or not PAP is displayed for vertical jumps [6]. The results of the current study indicate that although a small magnitude of PAP was displayed, larger muscle size (i.e., CSA) may not have been a positive contributing factor. It is probable that other characteristics, such as the nervous system or participant’s fiber type, contributed to an enhanced performance.

The current study displayed trivial relationships between strength measures and maximum PAP. These findings are in contrast with previous literature that noted small-to-large relationships between absolute muscular strength and PAP [14,15,16]. However, it should be noted that larger relationship magnitudes have been displayed between relative strength and PAP [17,18,19]. As indicated by our follow-up analysis, the relationship magnitudes between relative back squat and COHS strength and maximum potentiation grew to small-to-moderate magnitudes. Thus, while the primary analysis of the current study required the use of absolute strength rather than relative strength units (i.e., relative to kilogram of body mass), it is possible that the latter may contribute to a greater extent to PAP.

As mentioned in the previous paragraph, a potential limitation of the current study was the use of absolute strength and power measurements instead of relative values when assessing the relationships between variables. However, it should be noted that the current study warranted the use of absolute values rather than relative due to the standard units of muscle CSA.

## 5. Conclusions

The current study displayed moderate-to-large relationships between VL and BF CSA, back squat and COHS strength measures, and SJ power output. In contrast, trivial relationships existed between strength and maximum potentiation, while small negative relationships existed between CSA and maximum potentiation. These findings provide further information regarding the relationships between muscle CSA, strength, power, and potentiation. It appears that VL and BF CSA may positively influence back squat and COHS strength. Thus, practitioners may consider implementing resistance training methods that improve VL and BF size in order to benefit back squat and COHS performance. Back squat and COHS strength may have a positive influence on SJ power output indicating that squatting variations, both full and partial, may benefit jumping performance. Our results also indicate that muscle CSA and absolute muscular strength may not have much of an influence on maximum SJ PP potentiation; however, it should be noted that relative strength may have a greater influence on maximum potentiation compared to absolute strength.

## Figures and Tables

**Table 1:** Descriptive muscle CSA, strength, power, and maximum potentiation data.

Characteristic	VL CSA (mm^2^)	BF CSA (mm^2^)	1RM BS (kg)	1RM COHS (kg)	SJ PP (W)	Max PAP (%)
Mean ± SD	32.5 ± 5.8	18.1 ± 3.5	164.7 ± 29.9	195.0 ± 28.2	4867.2 ± 638.4	3.9 ± 3.6

Notes: VL = vastus lateralis; BF = biceps femoris; CSA = cross-sectional area; mm^2^ = millimeters squared; 1RM = one repetition maximum; BS = back squat; COHS = concentric-only half-squat; kg = kilograms; SJ = static jump; PP = peak power; W = watts; Max PAP = maximum potentiation.

**Table 2:** Relationships between muscle CSA, strength, power, and maximum potentiation.

Characteristic	VL CSA	BF CSA	1RM BS	1RM COHS	SJ PP	Max PAP
VL CSA	1.000					
BF CSA	0.669 *	1.000				
1RM BS	0.643 *	0.643 *	1.000			
1RM COHS	0.625 *	0.462	0.897 *	1.000		
SJ PP	0.396	0.683 *	0.548 *	0.407	1.000	
Max PAP	−0.229	−0.239	−0.013	0.149	−0.297	1.000

Notes: VL = vastus lateralis; BF = biceps femoris; CSA = cross-sectional area; 1RM = one repetition maximum; BS = back squat; COHS = concentric-only half-squat; SJ = static jump; PP = peak power output; Max PAP = maximum potentiation; * = statistically significant relationship (*p* ≤ 0.05).
